# Assessment of *Helicobacter pylori* cytotoxin-associated Gene A (Cag A) protein and its association with ferritin and vitamin B12 deficiencies among adult healthy asymptomatic residents in Sharjah, United Arab Emirates

**DOI:** 10.1016/j.heliyon.2024.e32141

**Published:** 2024-05-29

**Authors:** Om Kolthoom M. Weisy, Reena A. Kedia, Ibrahim Mahmoud, Raed O. Abu Odeh, Bashair M. Mussa, Salah Abusnana, Sameh S.M. Soliman, Jibran Sualeh Muhammad, Mohamad Hamad, Rose Ghemrawi, Ghalia Khoder

**Affiliations:** a Department of Pharmaceutics and Pharmaceuticals Technology, College of Pharmacy, University of Sharjah, Sharjah, United Arab Emirates; b Research Institute for Medical & Health Sciences, University of Sharjah, United Arab Emirates; c Department of Family and Community Medicine and Behavioral Sciences, College of Medicine, University of Sharjah, Sharjah, United Arab Emirates; d Department of Medical Laboratory Sciences, College of Health Sciences, University of Sharjah, Sharjah, United Arab Emirates; e Department of Basic Medical Sciences, College of Medicine, University of Sharjah, Sharjah, United Arab Emirates; f Diabetes and Endocrinology Department, University Hospital Sharjah, Sharjah, United Arab Emirates; g Clinical Science Department, College of Medicine, University of Sharjah, Sharjah, United Arab Emirates; h Department of Medicinal Chemistry, College of Pharmacy, University of Sharjah, Sharjah, United Arab Emirates; i College of Pharmacy, Al Ain University, Abu Dhabi, United Arab Emirates; j AAU Health and Biomedical Research Center, Al Ain University, Abu Dhabi, United Arab Emirates

**Keywords:** *H. pylori*, CagA, Vitamin B12, Ferritin, ELISA, Gastric cancer, Sharjah, UAE

## Abstract

The United Arab Emirates (UAE) serves as an effective epidemiological site for assessing *Helicobacter pylori* (*H. pylori*) infection due to its diverse population. However, comprehensive studies on the prevalence of *H. pylori* in the UAE are notably scarce. In depth prevalence studies are needed as a preventive measure against gastric cancer and other emerging extra gastric diseases associated with *H. pylori* infection. Aim: This study aimed to assess *H. pylori* infection and its virulent oncoprotein, the Cytotoxin-Associated Gene (Cag A) and its association with ferritin and vitamin B12 deficiencies. **Methods**: The study was conducted on 1094 healthy asymptomatic volunteers residents in the Sharjah Emirate, UAE. Enzyme-linked immunosorbent assay (ELISA) was performed to assess *H. pylori* infection using *H. pylori* antibodies (IgG), and detection of CagA protein using Cag A antibody (IgG) in the human serum. Ferritin and vitamin B12 serum levels were assessed and correlated to *H. pylori* infection. **Results**: This study focuses mainly on the assessment of *H. pylori* and its virulent factor CagA, in relation to vitamin B12 and ferritin deficiencies. Remarkably, 49.6 % of the participants were detected positive for *H. pylori*, with over half of these cases involving CagA positive strains. Notably, among Emirati participants, 76.11 % of those with *H. pylori* infection were CagA positive. Statistical analysis revealed a significant correlation between *H. pylori*, CagA level, and ferritin/vitamin B12 deficiencies. **Conclusion**: These findings emphasize the importance of timely detection and eradication of *H. pylori* not only as a preventive strategy against gastric cancer but also as an effective strategy to rescue the adverse effects from ferritin and vitamin B12 deficiencies, thereby improving the overall health outcomes of individuals affected by *H. pylori* infection.

## 1 Introduction

*Helicobacter pylori* (*H. pylori*) is a microaerophilic, Gram-negative bacillus that infects approximately half of the population, exhibiting significant geographic variability in prevalence [[1], [2], [3], [4]]. *H. pylori* is primarily transmitted through oral-oral or fecal-oral routes within families, especially in environments with poor sanitation and hygiene practices [3,5]. The prevalence of *H. pylori* is usually high in developing countries (85–95 %) compared to developed countries (30–50 %) [2,3,6]. Despite advancements in sanitation and eradication methods, the epidemiology of *H. pylori* infection continues to exhibit a significant increase. This prevalence remains particularly high in developing countries, where it is influenced by socioeconomic status and hygiene levels. Currently, 4.4 billion people worldwide are estimated to be have *H. pylori* infection [2].

*H. pylori* infection is typically acquired during childhood and often remains asymptomatic. It can persist throughout life if not treated with antibiotics and progress to various gastric diseases including chronic and acute gastritis, peptic and duodenal ulcers, gastric cancer (GC) and mucosa-associated lymphoid tissue (MALT) lymphoma [7,8]. Furthermore, several controversial studies have also demonstrated a link between *H. pylori* and iron-deficiency anemia, ferritin deficiency, vitamin B12 deficiency, and certain cases of idiopathic thrombocytopenic purpura [[9], [10], [11], [12], [13], [14], [15], [16]].

Among gastric and extra gastric diseases associated to *H. pylori*, GC remains the most serious disease attributed to this bacterium. Despite declining incidence rates, the global burden of GC is projected to increase by 62 % by 2040 [17]. In the Arab region, including the United Arab Emirates (UAE), the estimated age-standardized incidence rates showed an incidence rate of 4.4 per 100 000 populations [18]. Several experimental and meta-analysis studies have attributed the gastric carcinogenesis of *H. pylori* to the cytotoxin-associated antigen A (CagA), one of the key virulence genes in *H. pylori* [[19], [20], [21], [22], [23], [24], [25], [26]]. The translocation of the oncoprotein CagA into gastric epithelial cells, facilitated by the Type IV Secretion System (T4SS), is associated with GC [19]. Due to this potent interactive mechanism, the World Health Organization (WHO) has classified *H. pylori* as a class 1 carcinogen and one of the strongest risk factors for GC and MALT lymphoma [27].

*H. pylori* strains are commonly categorized as either CagA-positive or CagA-negative. The presence of CagA is frequently associated with mucosal inflammation and severe gastrointestinal conditions, such as peptic ulcers and GC [28]. Notably, approximately 30–40 % of Western *H. pylori* strains are CagA-negative, in contrast to nearly all East Asian *H. pylori* isolates, which are CagA-positive [29,30].

The incidence of GC is closely linked to the global prevalence of *H. pylori* [31,32]. Screening and eradication of *H. pylori* are cost-effective strategies that can significantly reduce the burden of GC in high-prevalence populations, offering potential to decrease GC-related mortality [[33], [34], [35], [36]].

In the latest global prevalence study conducted in 2018, data on the prevalence of *H. pylori* in the United Arab Emirates (UAE) were conspicuously missing among the 62 countries examined. The absence of informative data on *H. pylori* in the UAE, highlights the need to accurately assess the prevalence of *H. pylori* infection in UAE. Addressing this knowledge gap would be invaluable in understanding the epidemiological landscape of *H. pylori* in the UAE and provide appropriate preventive and management strategies.

Since *H. pylori* is well known by its genetic diversity and geographic variability, UAE constitutes a relevant site to conduct related epidemiological studies due to the multi nationalities and ethnicities residing in the country. Over the past three decades, 34 studies concerning *H. pylori* infection in the UAE have been conducted, according to PubMed data. However, only a limited number of these studies have assessed the *H. pylori* in asymptomatic subjects [[37], [38], [39]]. A recent pilot study conducted in 2019 found that 41 % of the UAE population was infected with *H. pylori*. The study identified a significant association between *H. pylori* infection and several sociodemographic factors and gastrointestinal characteristics of the participants [39]. Despite the intriguing nature of the findings and their novelty, it is crucial to acknowledge that the study was limited by its small sample size, consisting of only 350 participants. Furthermore, the investigation failed to assess key virulence factors, notably the oncoprotein CagA, as well as important clinical parameters such as ferritin and vitamin B12 levels, which are suggested as clinical outcomes of *H. pylori* infection. Growing evidence suggests a potential association between *H. pylori* infection, ferritin and vitamin B12 deficiencies [[40], [41], [42], [43], [44], [45], [46], [47], [48], [49]].

Since implementation of effective eradication strategies necessitates accurate and updated information regarding the local prevalence of *H. pylori*, the current study was conducted. Due to the significant global differences in the prevalence of *H. pylori* infection and GC, it is crucial for each country to evaluate the necessity of implementing a national screening and treatment program for *H. pylori*. In the case of UAE, comprehensive evaluation is warranted to determine the cost-effectiveness of such a program before its nationwide implementation. Up to date, there is no enough comprehensive national studies assessing *H. pylori* and its associated factors. Hence, the primary aims of this study are to assess *H. pylori* infection and its oncoprotein CagA on a large screen population representative of the different ethnicities residing in the UAE including the Emirati nationals. The secondary aim is to investigate the association between *H. pylori*, CagA seropositivity, ferritin and vitamin B12 deficiencies.

## 2 Materials and methods

### Ethical Statement

The study received review and approval from the Research and Ethics Committee of the University of Sharjah under reference number REC-17-04-17-01. The study involved the collection of sociodemographic data from healthy volunteer participants, which was recorded and handled with utmost confidentiality. Prior to participating in the study, all volunteers provided their informed consent by signing a consent form. These measures were taken to ensure that the study adhered to ethical guidelines and protected the privacy of the participants.

### 2.1 Study design, sample size and sample preparation

The cross-sectional study was conducted from January 2022 to January 2023. During this period, 1094 serum samples were sequentially collected from healthy asymptomatic volunteers at the Sharjah Municipality Public Health Clinic (SMPHC).

The sample size was determined using an online sample size calculator (https://www.calculator.net/sample-size-calculator.html). The calculation used a 95 % confidence level, 3 % margin of error, 40 % estimated prevalence from a previous study, and a population of 11 million [50]. Initially, 1025 participants were suggested, but 1094 were included to account for potential data exclusions.

The study included participants from both sexes with a mean age of 40.1 years (±14.59). Serum samples were collected using serum separator tubes (SST). The samples were permitted to clot for 2 h at room temperature or overnight at 4 °C, followed by centrifugation at 1000×*g* for 15 min. Approximately 1000 μL of serum were collected and were transported without delay to Research Institute for Medical & Health Sciences (RIMHS) laboratory, University of Sharjah, where aliquots of fresh serum samples were stored at −20 °C or −80 °C for further screening. Socio-demographic data (gender, age, nationality, and occupation) were obtained in parallel with the collected serum samples. The purpose of the study was explained briefly to study participants prior to sample collection by the recruited research assistant. Serum samples were processed for further steps only after a signed consent from by the study participants. To ensure the study was accessible and comprehensible to participants of all nationalities, the consent form was prepared in both Arabic and English. Participants from 38 nationalities were involved in the study. For analysis purpose, participants were grouped into four main ethnicities residents in UAE as follows: Asian (N = 766, 70 %); Arab (N = 275, 25.1 %); African (N = 444.1 %) and Western (N = 9, 0.8 %). Arabs were mainly from the following countries: UAE, Jordan, Iraq, Syria, Egypt, and Palestine. Asians were mainly from Pakistan, India, Bangladesh, Nepal, Philippine, and Indonesia. Africans were mainly from Sudan, and Ethiopia. A summary of the detailed socio-demographic profiles of the study participants is provided in Table 1.

**Table 1:** Socio-demographic profiles of the study participants according to gender, age, ethnicity, occupation, *H. pylori* infection and CagA status, vitamin B12 status (pmol/L) and ferritin status (ng/ml), n = 1094.

Variable	n (%)/Mean (SD)
**Gender**	
Female	715 (65.4)
Male	379 (34.6)
**Age, years**	
Mean (SD)	40.10 (14.59)
19–29	297 (27.1)
30–39	334 (30.5)
40–49	213 (19.5)
50–59	117 (10.7)
≥60	133 (12.2)
**Ethnicity**	
Arab	275 (25.1)
Asian	766 (70.0)
African	44 (4.1)
Western	9 (0.8)
**Occupation**	
Professional	221 (20.2)
Laborer	416 (38)
Food industry	102 (9.3)
Housewife/maid wife	172 (15.7)
Student	36 (3.3)
Unemployed	147 (13.5)
**Hp infection status**	
Negative	551 (50.4)
Positive	543 (49.6)
**Vitamin B12 status, pmol/L**	
Mean (SD)	350 (326.8)
Normal	706 (64.6)
Deficient	388 (35.4)
**Ferritin status, ng/ml**	
Mean (SD)	202.8 (220)
Normal	1026 (93.8)
Deficient	68 (6.2)
**CagA status**	
Negative	776 (70.9)
Positive	318 (29.1)

SD= Standard Deviation.

For the exclusion and inclusion criteria of this study, participants were disqualified if they 1) experienced recent gastrointestinal disturbances, 2) had undergone any antimicrobial treatments within the past two weeks, or 3) had received *H. pylori* treatment within the last six months. These criteria ensured that the study focused on predominantly healthy, asymptomatic individuals to accurately assess the prevalence of *H. pylori*. Participants who were receiving treatments for ferritin or vitamin B12 deficiencies were also excluded. The study population comprised healthy expatriate residents of various nationalities and occupations, as well as Emirati participants aged 19 years and older, residing in the UAE.

### 2.2 PREMIER *H. pylori* serum test

The PREMIER *H. pylori* (Ref: 606096, Meridian BIOSCIENCE, USA) is an enzyme immunoassay (EIA) designed for the qualitative detection of IgG antibodies to *H. pylori* in human serum in vitro. This assay uses a sonicated H. pylori bacterial cell lysate that is coated onto plastic microwells. Patient serum samples were diluted and incubated in these wells at temperatures between 19 °C and 27 °C. Following the incubation, the wells were washed to remove unbound substances, and then a peroxidase-conjugated monoclonal antibody specific to human IgG was added. The plates were subsequently reincubated to allow for binding. After a second washing step to eliminate any unbound antibody conjugate, PREMIER Substrate I was introduced to the wells to facilitate color development, which occurs in response to the presence of the enzyme-linked antibody. To halt the reaction and stabilize the color, PREMIER Stop Solution I was added. The reactions were observed either visually or spectrophotometrically by reading the absorbance at 450 nm within 10 min of adding PREMIER Stop Solution I. Visual reading yields a negative result when the sample appears colorless and a positive result when the sample shows a definite yellow color. Spectrophotometric measurements at a single wavelength of 450 nm were interpreted as negative if the optical density (OD450) is less than 0.12 and positive if it is equal to or greater than 0.12. For optimal performance, all samples and controls were assayed in duplicate.

### 2.3 CagA ELISA test

The Human cytotoxin-associated protein (CagA) antibody (IgG) ELISA kit (Cat. # CSB-EQ027541HU; Cusabio, Houston, TX, USA) was utilized to quantify the IgG levels against the CagA protein in serum. This assay employed a pre-coated antigen microtiter plate to measure the expression levels. The ELISA kit includes a microtiter plate pre-coated with an antigen specific to CagA antibody. Samples containing potential antibodies are added to the wells. Anti-human IgG conjugated with Horseradish Peroxidase (HRP) is also added, which will bind to any antibodies specific to the pre-coated antigen. After incubation, the wells are washed to remove any unbound reagents, ensuring that only the antibodies bound to the pre-coated antigen remain in the wells. A substrate solution is introduced, which reacts with the HRP enzyme linked to the bound antibodies, producing a color change. The intensity of this color is directly proportional to the amount of antibody bound to the antigen in the wells. The reaction is stopped by adding a stopping solution, and the color intensity is measured. This intensity correlates with the concentration of the human cytotoxin-associated protein antibody in the sample. Samples and all reagents were prepared as previously described [51].

A negative result is obtained when the optical density (OD) value of the sample well is less than 2.1 times the OD value of the negative control. In contrast, a positive result is obtained when the OD value of the sample well is equal to or greater than 2.1 times the OD value of the negative control. For optimal performance, same samples and controls were assayed in duplicate. All samples stored at −20 °C or −80 °C were tested within 1 month and 2 months, respectively to avoid loss of bioactivity and contamination. The assay present high sensitivity and specificity for detecting CagA antibody and does not present any significant cross-reactivity or interference between CagA and analogues.

### 2.4 Vitamin B12 ELISA test

Vitamin B12 ELISA kit (REF: EIA- 5848, DRG International, USA) was used to measure the concentration of serum vitamin B12. Essential reagents such as an antibody, enzyme-antigen conjugate, and native antigen are used. Initially, a biotinylated antibody is mixed with a serum that contains the antigen, prompting a binding reaction. Following a brief incubation period, an enzyme conjugate is introduced, leading to a competitive reaction with the antigen for a limited number of antibody binding sites. Concurrently, the biotin on the antibody reacts with the streptavidin on the microwell, which facilitates the separation of the antibody-bound fraction through decantation or aspiration. The level of enzyme activity in this fraction is inversely related to the concentration of the native antigen. By comparing this activity against a dose-response curve created using serum references with known antigen concentrations, the antigen level in an unknown sample can be accurately determined. All samples and reagents were prepared following the manufacturer's protocol. Absorbance was read at 450 nm within 15 min of adding the stop solution. Samples suspected of concentrations higher than 2000 pg/mL were diluted to 1:5 and 1:10 with Vitamin B12 calibrator and re-assayed. Based on the manufacturer reference values, a serum vitamin B12 concentration below 148 pmol/L in adults below or equal to 60 years old, was considered as vitamin B12 deficiency condition. While a serum level below 81 pmol/L in adults above 60 years was considered as a vitamin B12 deficient condition. For optimal performance, all samples and controls were assayed in duplicate.

### 2.5 Ferritin ELISA test

The Ferritin ELISA kit (REF: EIA-4408, DRG International, USA) operates on the principle of simultaneous binding of human ferritin to two monoclonal antibodies—one immobilized on microwell plates and the other conjugated with horseradish peroxidase (HRP). Samples and reagents were prepared in accordance with the manufacturer's instructions. Following incubation, bound/free separation was achieved through a straightforward solid-phase washing. Subsequently, the TMB substrate was added. After allowing sufficient time for color development, the enzyme reaction was halted, and absorbance was measured. Ferritin concentration in the sample was calculated using a series of standards, with color intensity being directly proportional to ferritin concentration. Absorbance values for the standards were plotted against their respective concentrations, and the mean absorbance value for each sample was determined based on this standard curve. The concentrations of the samples in ng/mL were obtained by interpolating their absorbance values on the standard curve. Based on the manufacturer reference values, a range of [6–180 ng/ml] and [8–350 ng/ml] were considered as a normal ranges of serum ferritin in premenopausal and post-menopausal women, respectively. A range of [20–400 ng/ml] was considered as a normal level of serum ferritin in men. An obtained concentration less than 6 ng/ml in women or 20 ng/ml in men was reported as a ferritin deficiency condition. For optimal performance, all samples and controls were assayed in duplicate.

### 2.6 Statistical analysis

Descriptive statistics were used to summarize the characteristics of the study participants. Continuous variables were reported as means with standard deviations (SD), while categorical variables were presented as frequencies and percentages. Pearson's chi-square test examined the relationship between *H. pylori* infection and categorical variables such as gender, age groups, ethnicity, occupation, vitamin B12 status, ferritin status, and CagA status. To identify factors associated with *H. pylori* infection, binary logistic regression analysis was conducted. A p-value of ≤0.05 was considered statistically significant. All analyses were performed using IBM SPSS Statistics for Windows, version 28.0 (IBM Corp., New York, USA).

## 3 Results

### 3.1 Socio-demographic profile of study participants

A total of 1094 volunteer subjects were included in the current epidemiological study (Table 1). Among participants, there were 715 (65.4 %) females and 379 (34.6 %) males. The participants' ages ranged from 19 to 70 years, with an average age of 40.10 ± 14.59 years. Among the studied age groups, participants aged between 19 and 39 years constituted the largest group (631, 57.6 %). While, the age group from 50 to 59 years constituted the fewest number of participants (117, 10.7 %). The ethnicity of the participants was distributed as follows: Arab (275, 25.1 %), Asian (766, 70.0 %), African (44, 4 %), and Western (9, 0.8 %). The ethnicity of the participants was distributed over 38 nationalities residents in UAE, The major nationalities of the participants were distributed as following: Indian (363, 33.2 %), Pakistani (175, 16 %), Emiratis (147, 13.4 %), Bangladeshi (101, 9.2 %), Filipino (58, 5.3 %), Egyptian (48, 4.4 %), Syrian (40, 3.7 %), Nepali (33, 3 %), Ethiopian (18, 1.64 %) and Sudanese (13, 1.2 %). While remaining minor nationalities represented around 8.96 % of the total participants. Only very few participants from the Western ethnicity participated in this study maybe because the emirate of Sharjah is usually hosted by more Arab and Asians residents compared to the other emirates.

In terms of occupation, it was distributed as following: employed as laborer (417, 38 %), employed as professional (221, 20.2 %), employed as maid wife and housewife (172, 15.7 %), employed as worker in food industry (102, 9.3 %), and student (36, 3.3 %). Non employed participants constituted 13.5 % (Table 1).

### 3.2 Assessment of *H. pylori* infection, CagA, vitamin B12 and ferritin

Analysis of the 1094 collected serum samples revealed that 543 individuals were infected with *H. pylori*, resulting in an estimated infection rate of 49.6 % (Table 1). Interestingly, among the total participants (n = 1094), 318 (29.1 %) were found infected with a CagA positive strain which accounts to 58.5 % from the total number of *H. pylori* positive participants (n = 543). Regarding the serum vitamin B12, 706 (64.6 %) of the participants showed a normal vitamin B12 levels, while 388 (35.4 %) showed vitamin B12 deficiency. The mean value of the vitamin B12 status of all participants was 350 ± 326.8 pmol/L. Similarly, most of the participants showed normal serum ferritin level (1026, 93.8 %) compared to only 68 participants (6.2 %) with ferritin deficiency. The mean value of the ferritin status of all participants was 202.8 ± 220 ng/ml.

### 3.3 Correlation of *H. pylori* infection with socio-demographic profiles, vitamin B12, and ferritin deficiencies

Bivariate analysis revealed significant differences in *H. pylori* infection based on gender, ethnicity, occupation, and ferritin deficiency (Table 2). Males were more prone to infection (376, 52.6 %) compared to females (167, 44.1 %), with a significant statistical difference (p = 0.007). No significant difference was found between *H. pylori* infection and age groups. The age groups from 19 to 29 and 50 to 59 presented the highest percentages of infection (53.9 % and 52.1 %, respectively).

**Table 2:** Bivariate analysis of *H. pylori* infection in relation to socio-demographic profiles, vitamin B12, and ferritin deficiencies among study participants (n = 1094).

Variables	Hp infection status, n (%)		P value
	Negative, n = 551	Positive, n = 543	
**Gender**			
Female	212 (55.9)	167 (44.1)	**0.007**
Male	339 (47.4)	376 (52.6)	
**Age, years**			
19–29	137 (36.1)	160 (53.9)	
30–39	170 (50.9)	164 (49.1)	
40–49	113 (53.1)	100 (46.9)	0.286
50–59	56 (47.9)	61 (52.1)	
≥60	75 (56.4)	58 (43.6)	
**Ethnicity**			
Arab	148 (53.8)	127 (46.2)	
Asian	382 (49.9)	384 (50.1)	
African	13 (29.5)	31 (70.5)	**0.002**
Western	8 (89.9)	1 (11.1)	
**Occupation**			
Professional	118 (53.4)	103 (46.6)	
Laborer	181 (43.5)	235 (56.5)	
Food industry	56 (54.9)	46 (45.1)	**0.011**
Housewife/maid wife	93 (54.1)	79 (45.9)	
Student	24 (66.7)	12 (33.3)	
Unemployed	79 (53.7)	68 (46.3)	
**Vitamin B12 status, pmol/L**			
Normal	364 (51.6)	342 (48.4)	0.394
Deficient	187 (48.2)	201 (51.8)	
**Ferritin status, ng/ml**			
Normal	540 (52.6)	486 (47.3)	**< 0.001**
Deficient	11 (16.2)	57 (83.8)	

The P value was determined using Pearson's chi-square test. Statistically significant P values are highlighted in bold.

**Fig. 1:**
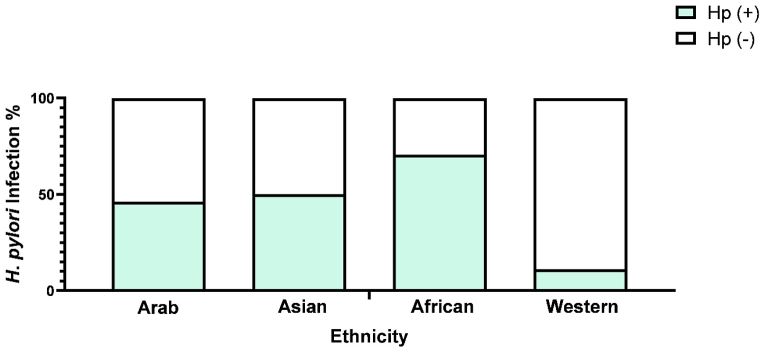
*H. pylori* infection (%) across the different study participants ethnicities.

Given the diverse ethnic composition of the UAE, it was important to examine which ethnic groups are more susceptible to *H. pylori* infection. The analysis revealed significant statistical difference in *H. pylori* infection rates among the different ethnicities (p = 0.002) (Table 2). The African ethnicity presented the highest *H. pylori* prevalence (31, 70.5 %) while Arab ethnicity presented the lower prevalence (127, 46.2 %). The Asian ethnicity presented a very close prevalence to Arab ethnicity (384, 50.1 %) and was ranked two among the tested ethnicities. Due to the small number of western participants, the obtained results were mostly neglected (Fig. 1).

**Fig. 2:**
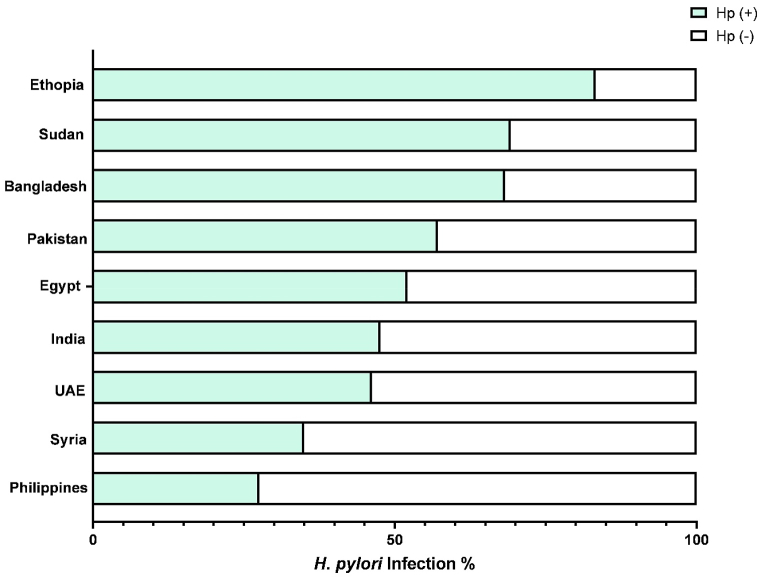
*H. pylori* infection (%) across the main study participants nationalities.

In terms of nationality, the Ethiopian, Sudanese, and Bangladeshi participants presented the highest percentage of *H. pylori* infection (83 %, 69.2 %, 68.3 % respectively). The lowest *H. pylori* prevalence was obtained among Filipino participants (27.6 %). Almost half (45.57 %) of the Emiratis subjects were found infected by *H. pylori* (Fig. 2). Therefore, the UAE nationals ranked among the second top highest in *H. pylori* prevalence among the tested Arab ethnicity.

A statistically significant difference was observed between *H. pylori* infection and participants' occupations, including professionals, laborers, food industry workers, housewives, students, and the unemployed (p = 0.011). Laborers exhibited the highest infection rate (235, 56.5 %), followed by professional workers (103, 46.6 %), housewives and maids (79, 45.9 %), food industry workers (46, 45.1 %), and students (12, 33.3 %) (Table 2).

Regarding vitamin B12 deficiency, 201 participants with *H. pylori* infection were found to be deficient in vitamin B12. Thus, the prevalence of vitamin B12 deficiency among *H. pylori*-positive participants was determined to be 51.8 %. Although 35.4 % of the study participants had vitamin B12 deficiency, and previous research has linked this deficiency to *H. pylori* infection, no statistically significant association was found between *H. pylori* infection and vitamin B12 deficiency among UAE residents. However, a significant difference was observed between *H. pylori* infection and ferritin deficiency (p < 0.001). Of the 68 participants with ferritin deficiency (6.2 % of the total study population), 57 (83.8 %) tested positive for *H. pylori* (Table 2).

Conversely, binary logistic regression analysis indicated that male participants are significantly more likely to be diagnosed with *H. pylori* infection compared to female participants (Odds Ratio (OR) 1.74, 95 % CI, 1.22–2.49, p = 0.002). Additionally, individuals of African descent were found to be more susceptible to the infection (OR 3.45, 95 % CI, 1.55–7.71, p = 0.002) compared to Arab and Asian ethnicities. In terms of occupation, laborers were more susceptible to the infection (Odds Ratio (OR) 1.41, 95 % CI, 1.01–1.99, p = 0.05) compared to professionals, food industry workers, housewives and maids, students, and the unemployed (Table 3). Interestingly, the ferritin deficiency was also found as risk factor for *H. pylori* infection on the tested population. Participants with ferritin deficiency were significantly more susceptible to *H. pylori* infection (OR 3.69, 95 % CI 2.39–5.69, p < 0.001) compared to those with normal ferritin levels.

**Table 3:** Binary logistic regression analysis for factors associated with *H. pylori* infection, n = 1094.

Variable	OR (95%CI)	P value
**Gender**		
Female	Reference	
Male	1.74 (1.22–2.49)	**0.002**
**Ethnicity**		
Arab	Reference	
Asian	0.89 (0.59–1.35)	0.586
African	3.45 (1.55–7.71)	**0.002**
Western	0.40 (0.08–1.96)	0.257
**Occupation**		
Professional	Reference	
Laborer	1.41 (1.01–1.99)	**0.050**
Food industry	0.87 (0.54–1.41)	0.573
Housewife/maid wife	1.12 (0.69–1.84)	0.644
Student	0.56 (0.25–1.24)	0.150
Unemployed	1.13 (0.65–1.94)	0.668
**Ferritin status, ng/ml**		
Normal	Reference	**< 0.001**
Deficient	3.69 (2.39–5.69)	

CI stands for Confidence Interval; P values were calculated using the binary logistic regression model. Statistically significant P values are highlighted in bold.

### 3.4 Correlation between CagA positivity and socio-demographic profiles, vitamin B12 and ferritin deficiencies

**Fig. 3:**
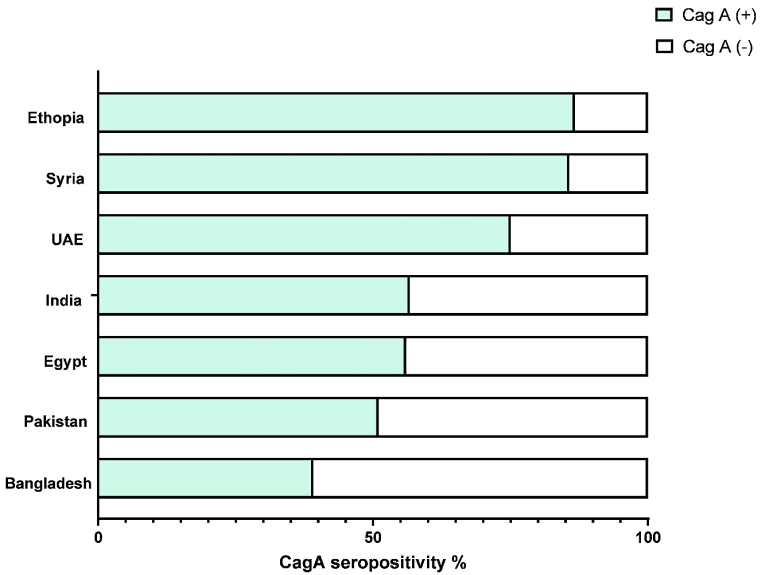
CagA seropositivity (%) among the different study participants nationalities.

Bivariate analysis showed that the oncoprotein CagA exhibited statistically significant differences across several sociodemographic characteristics including gender, age, and ethnicity, vitamin B12 and ferritin levels (Table 4). Female participants were more likely to be CagA seropositive (121, 72.5 %) compared to male participants (197, 52.4 %), with this difference being statistically significant (p < 0.001). Unlike *H. pylori* status, CagA status showed a statistically significant difference across different age groups (p < 0.001). CagA seropositivity was found to increase significantly with age. Participants aged 50 years presented the highest prevalence in CagA (>75 %). Additionally, a statistically significant difference was observed between CagA seropositivity and ethnicity (p < 0.001). African ethnicity presented the highest prevalence with CagA *H. pylori* strain (87.1 %) followed by Arab and Asian ethnicities (69.3 % and 52.6 %, respectively). Among all the included nationalities, it's interesting to mention that similarly to the *H. pylori* status, Ethiopians presented the highest prevalence with CagA (72.2 %). Even though, Indian, Pakistani, and Bangladeshi presented a high *H. pylori* prevalence, this was not reflected at the CagA level where they presented the lowest prevalence in CagA (27 %, 29 %, 26.7 % and 29.16 %, respectively). However, among the Emirati participants, its worthy to mention that among the 45.57 % *H. pylori* positive participants, 76.11 % were found infected with CagA positive *H. pylori* strain and therefore ranked as second after the Syrian Arab participants (Fig. 3). The other nationalities were neglected due to the small representative sample size.

**Table 4:** Factors associated with CagA seropositivity among *H. pylori* infected patients, n = 543.

Variable	Cag A status, n (%)		P value
	Negative, n = 225	Positive, n = 318	
**Gender**			
Female	46 (27.5)	121 (72.5)	**< 0.001**
Male	179 (47.6)	197 (52.4)	
**Age, years**			
19–29	91 (56.9)	69 (43.1)	
30–39	71 (43.3)	93 (56.7)	
40–49	35 (35.0)	65 (65.0)	**< 0.001**
50–59	14 (23.0)	47 (77.0)	
≥60	14 (24.1)	44 (75.9)	
**Ethnicity**			
Arab	39 (30.7)	88 (69.3)	**< 0.001**
Asian	182 (47.4)	202 (52.6)	
African	4 (12.9)	27 (87.1)	
**Vitamin B12 status, pmol/L**			
Normal	192 (56.1)	150 (43.9)	**< 0.001**
Deficient	33 (16.4)	168 (83.6)	
**Ferritin status, ng/ml**			
Normal	209 (43)	277 (57)	**0.013**
Deficient	16 (28)	41 (72)	

The P value was determined using Pearson's chi-square test. Statistically significant P values are highlighted in bold.

**Fig. 4:**
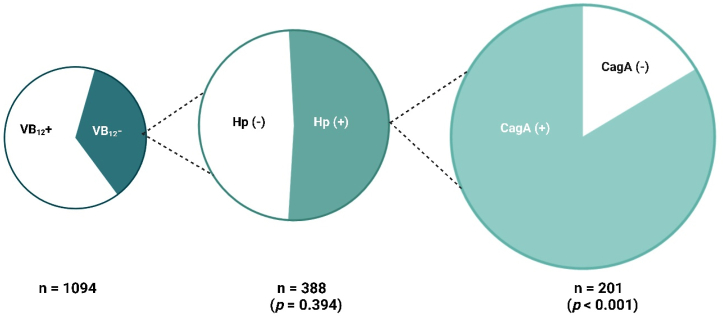
Association of vitamin B12 deficiency with *H. pylori* and CagA seropositivity.

In contrast to *H. pylori* status, no significant statistical association was found between CagA seropositivity and participants' occupations (Table 4). However, a statistically significant difference was observed between the oncoprotein CagA and both vitamin B12 deficiency (p < 0.001) and ferritin deficiency (p = 0.013) (Table 4). Among the 201 *H. pylori*-infected participants with vitamin B12 deficiency, 168 (83.6 %) were infected with the CagA-positive *H. pylori* strain, while 33 (16.4 %) were infected with the CagA-negative strain (Fig. 4). Fig. 4 illustrates the association between vitamin B12 deficiency and seropositivity for *H. pylori* and its virulence factor, CagA, in 1094 study participants. It highlights that 35.4 % (388 participants) had vitamin B12 deficiency, of whom 51.8 % (201 participants) tested positive for *H. pylori*. Among these *H. pylori* positive individuals, 83.6 % (168 participants) were also CagA positive. The figure notes a statistically significant correlation between CagA seropositivity and vitamin B12 deficiency (*p* < 0.001).

**Fig. 5:**
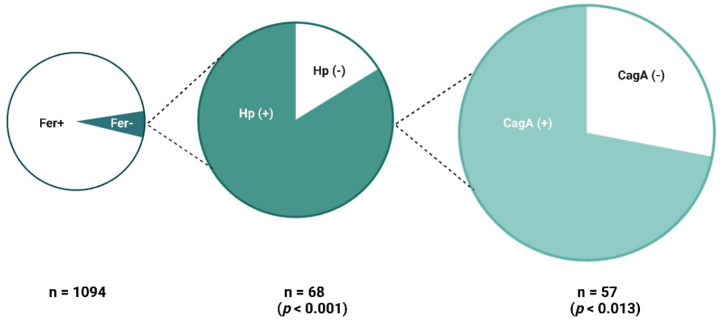
Association of ferritin deficiency with *H. pylori* and CagA seropositivity.

Similarly, among the 57 ferritin deficient *H. pylori* infected participants, 41 (72 %) were infected with the carcinogenic strain of *H. pylori* compared to 16 (28 %) who were infected with a noncarcinogenic *H. pylori* strain (Fig. 5). Fig. 5 illustrates the relationship between ferritin deficiency and seropositivity for *H. pylori* and CagA among 1094 participants. It shows that 6.2 % (68 participants) had ferritin deficiency, of which 83.8 % (57 participants) tested positive for *H. pylori*. Among these *H. pylori* positive participants, 72 % (41 participants) were also CagA positive. The associations between ferritin deficiency and both *H. pylori* infection and CagA seropositivity were statistically significant, with *p*-values of less than 0.001 and 0.013, respectively.

## 4 Discussion

Epidemiological studies play an essential role in the implementation of eradication and preventive strategies within the overall management of *H. pylori*, including identification of specific risk factors and prevention of GC. Based on the latest estimates released by GLOBOCAN, in 2020, GC is classified as the fourth leading cause of cancer-related mortality [52]. It was reported in over one million new cases in 2020 alone and was the cause of approximately 769 000 deaths [52]. In the Arab region, including the UAE, the estimated incidence rates showed an incidence rate of 4.4 per 100 000 populations [18]. Around 90 % of GC cases are linked to *H. pylori* infection [50]. In 2018, 812 000 cases were reported, making up about 37 % of all cancers caused by chronic infections, highlighting *H. pylori* as the leading carcinogenic pathogen [53]. The lifetime risk of developing GC in those infected with *H. pylori* ranges from 1 % to 5 %, influenced by ethnicity and environmental factors [33,54,55]. Several studies have attributed the gastric carcinogenesis of this bacteria to the cytotoxin associated antigen A (CagA), one of the key virulence genes in *H. pylori* [[20], [21], [22], [23], [24], [25]]. The oncoprotein CagA protein is first injected into gastric epithelial cells via T4SS. Following its translocation into the host gastric cell, CagA plays an important role in the development of local neoplasia [[56], [57], [58], [59], [60]]. Additionally, reports have indicated associations between *H. pylori* infection and diseases affecting organs beyond the stomach [8,61]. Growing evidence suggests a potential association between *H. pylori* infection, ferritin and vitamin B12 deficiencies [[40], [41], [42], [43], [44], [45], [46], [47]]. However, it is important to note that the evidence supporting these connections is limited, inconsistent, and inconclusive. While only few observational studies have demonstrated a correlation between the oncoprotein CagA and the vitamin B12 deficiency [43], none of the clinical studies have investigated the association between the CagA oncoprotein and the ferritin deficiency. Based on the strong implication of *H. pylori* and its oncoprotein CagA in GC as well as in other extra-gastric diseases, it become crucial to eradicate this bacterial infection as a preventive measure toward GC especially in countries where the prevalence of both GC and *H. pylori* is high. To this purpose, the 2015 Kyoto *H. pylori* conference and the 2019 Taipei consensus strongly endorses the eradication of *H. pylori* in asymptomatic patients as a preventive measure from GC [33,35,36]. Furthermore, the consensus proposes that populations with an elevated risk of GC contemplate the implementation of widespread screening initiatives coupled with *H. pylori* eradication efforts [1]. It is now widely accepted that the incidence rates of GC are closely linked to the global prevalence of *H. pylori* infection [31,32]. Research indicates that screening and eradicating *H. pylori* is a relatively cost-effective strategy for lowering the incidence of GC and peptic ulcers, particularly in populations with high infection rates. This approach shows significant potential for reducing mortality related to GC. In a recent study, Chen et al. assessed *H. pylori* infection among asymptomatic individuals in China. Their findings indicated that this approach was both more cost-effective and more efficient than not screening, in terms of preventing GC, peptic ulcers, and non-ulcer dyspepsia [62]. Therefore, to effectively implement eradication strategies, it is essential to have current data on the local prevalence of *H. pylori* and its related factors. Given the significant global variation in the prevalence of *H. pylori* infection and GC, each country should assess the need for a national *H. pylori* screening and treatment program.

Taking together the emerging evidence of association between *H. pylori* and its oncoprotein CagA and Ferritin and Vitamin B12 deficiency and the lacking studies related to ferritin and vitamin B12 in UAE, this prompted us to conduct this present study.

The success of epidemiological study relies in priority on the sample size and the employed diagnostic tool. In our study, we have included 1094 participants from the major four ethnicities resident in the UAE. This is the first large *H. pylori* assessment study conducted in UAE. The suggested sample size was initially 1025 participants; however, in our study, we included 1094 participants to account for any equivocal data that may have been removed during the study. Regarding diagnostic tests, each method for detecting *H. pylori* serves different purposes and has its own limitations [63,64]. The gold standard for *H. pylori* detection is endoscopic examination combined with gastric biopsy culture, which is known for its high reliability. However, this approach is expensive and logistically challenging for epidemiological studies because it requires specialized medical facilities and equipment [5,65]. Molecular techniques like PCR offer the advantage of testing antimicrobial susceptibility but still rely on the availability of gastric biopsy. Considering the limitations of previous tests, serological tests remain the most suitable and widely used tool for epidemiological screening [66,67]. Unlike other diagnostic tests, serological tests are cheap, simple, rapid and do not require an endoscopic examination or bacterial culture [68]. Furthermore, they are reliable and robust enough to be extensively used in several clinical epidemiological studies [1,[69], [70], [71], [72]]. Our study selected the PREMIER *H. pylori* serology test largely because it does not require invasive procedures. The PREMIER *H. pylori* test is designed for the in vitro qualitative detection of IgG antibodies to *H. pylori* in human serum. Although serological tests require a phlebotomist and are not as accurate as some other tests, they have been widely and successfully used in epidemiological studies [63,64]. One of the major advantages of serological testing is that they can detect past and active *H. pylori* infections [73]. Even though, detection of past infection could be a disadvantage of the test when used for eradication confirmation, in our current research study, it was an advantage because our aim was to assess asymptomatic participants with both current infection and past infection. Unlike other stool tests, the PREMIER *H. pylori* serum test is the only test not influenced by the current Proton Pump Inhibitor (PPI) intake [1,[69], [70], [71]]. It is also noteworthy that numerous studies have demonstrated that serological tests, when compared to the gold standard urea breath test (UBT) for *H. pylori* detection, yield reliable and comparable results [72]. Furthermore, serological tests seem ideal for epidemiological studies because they may provide additional data on the virulence of the *H. pylori* by detecting antibodies against CagA antigen [74]. To this purpose, the human CagA immunoglobulin G (IgG) ELISA test has been also selected to assess the CagA seropositivity among participants. In detecting *H. pylori* by ELISA, it is generally recommended to use two different antibody types for increased specificity and accuracy. The most used immunoglobulins are the IgG and IgM antibodies in the ELISA test. IgG antibodies provide high sensitivity and are suitable for detecting both current and past *H. pylori* infections. In our study, we have only used one of the immunoglobulin IgG to detect *H. pylori* and CagA due to the large sample size, the duplicated samples, and the restricted budget for this study. This is one of the limitations to be considered in further epidemiological studies. Additionally, the study's geographic scope was confined to Sharjah due to challenges in obtaining multi-regional ethical approvals, limiting the diversity of the population sample. We recognize that extending the study to include other emirates would enhance the generalizability of our findings across the diverse ethnic landscape of the UAE.

The current study was conducted to address the limited availability of data concerning *H. pylori*, its oncogenic virulence factors CagA, and associated risk factors, with the aim of contributing new insights to this field of research. Previous investigations conducted in the UAE have reported varying prevalence rates of *H. pylori* infection, with outcomes influenced by the specific focus of each study. Adeyemi et al., in 1992 and Zaitoun et al., in 1994 observed a prevalence of 90 % among dyspeptic patients [75,76]. A study conducted by Albawardi et al., in 2013, investigating complications of sleeve gastrectomies has found *H. pylori* infection in 44 % of patients [77]. In 2007, Bener et al. explored the link between type 2 diabetes mellitus and *H. pylori* infection, discovering a prevalence of 76.7 % among diabetic subjects, compared to 64.8 % in non-diabetic subjects [78]. Earlier, Bener et al. conducted two studies in 2002 and 2006 that examined the seroprevalence of *H. pylori* in asymptomatic Emirati patients [37,38]. The initial prospective study, which included 151 subjects from both farming and non-farming backgrounds, reported *H. pylori* prevalence rates of 74.2 % using IgG antibodies and 51 % using IgA antibodies, with no significant difference in prevalence between farmers and non-farmers [38]. Additionally, a study focusing on asymptomatic individuals from low socioeconomic backgrounds revealed *H. pylori* prevalence rates of 78.4 % in industrial workers compared to 64.3 % in control workers, showing a statistically significant difference between the two groups [37]. More recently, a 2019 study reported a prevalence rate of 41 % among a restricted population of 350 participants, including both children and adults. This study identified significant associations between *H. pylori* infection, some socio-demographic, and gastrointestinal characteristics of the patients [39]. However, the study's scope was limited to a specific population and did not assess *H. pylori* virulence factors and other risk factors such as ferritin and vitamin B12 deficiency. Even though previous referenced studies have contributed to the understanding of *H. pylori* prevalence in the UAE and have reflected the diversity of prevalence rates across different study populations and contexts, they were not representative of the UAE population. They primarily focused on dyspeptic individuals or specific occupational groups [37,38]. In summary, none of these studies considered the asymptomatic multinational residents in the UAE neither the *H. pylori* carcinogenic virulent factor CagA, and their association with vitamin B12 and ferritin deficiencies. In our study, approximately half of the healthy volunteered subjects (49.6 %) were found *H. pylori* positive while 58.5 % were CagA positive. Our findings were comparable to Arab neighboring country such as Oman (49.1 %) but lower than Saudi Arabia [2], Bahrain [79], Kuwait [80], Jordan and Iraq [81,82]. Compared to African countries such as Egypt and Ethiopia, our results have showed also lower prevalence [6,[83], [84], [85], [86]]. Compared to Asian countries such as India and Bangladesh, *H. pylori* prevalence in adults was estimated to 90 % and 88 %, respectively. The high prevalence of *H. pylori* in Asian countries was not reflected on the Asian ethnicity residing in the UAE. However, it was mainly reflected on the African ethnicity. In summary, despite the multi-nationalities and the multi-ethnicities of the UAE residents, the prevalence of *H. pylori* remains low compared to Arab, Asian and African countries and aligned with the declining global prevalence of *H. pylori* from 55 % to 43 % during the 2014–2020 [2,32].

In the current study, the prevalence of *H. pylori* and its oncoprotein CagA was assessed in asymptomatic healthy adults residing in UAE. Additionally, we examined the associations between *H. pylori* infection and socio-demographic characteristics such as age, gender, ethnicity, occupation, as well as clinical parameters like ferritin and vitamin B12 deficiencies. The findings of the current study will provide a valuable and strong foundation for future implementation of national program related to the prevention of GC associated to *H. pylori* infection as well as to urge the necessity to conduct future *H. pylori* antibiotic resistance prevalence studies.

Our findings indicated a significant difference in *H. pylori* and CagA positivity between genders. Male participants exhibited a higher prevalence of *H. pylori* infection (p = 0.007), whereas female participants were more frequently infected with CagA-positive strains (p < 0.001). The gender disparity in *H. pylori* infection requires further research to understand how sex affects the acquisition and persistence of the infection. Further investigations are required to explore whether estrogen hormone could affect the infectivity with the oncoprotein CagA. Regarding age, our findings indicated no significant correlation between the prevalence of *H. pylori* infection and different adult age groups. However, there was a statistically significant difference in CagA positivity among age groups (p < 0.001), particularly in individuals over 50 years old. These results align with findings from other studies [87]. In terms of ethnicity susceptibility to *H. pylori* infection and CagA seropositivity, the African ethnicity was most prone to the infection and particularly to the CagA strains. These findings align with a previous cohort study indicating that Black and African Americans are more susceptible to GC compared to other ethnic groups in the US. This increased risk is attributed to lower rates of *H. pylori* testing and eradication, leading to a higher likelihood of chronic infection [88].

Furthermore, Brown et al., has reported through a systematic review that among five studies investigating *H. pylori* CagA prevalence based on race, four reported a higher *H. pylori* prevalence among Blacks and Hispanics in comparison to whites. In these studies, the prevalence of CagA varied widely, ranging from 19 % to 77 % among whites, 62 %–90 % among Blacks, and 64 %–74 % among Hispanics [89,90]. In the Arab ethnicity, Emirati participants presented one of the highest prevalence of *H. pylori* (45.57 %). This could be explained by the close interaction between the Emiratis population and Indians through their lifestyle. It is very popular in the UAE to recruit cooks from India because Indian food is one of the most popular cuisines among local dishes. Furthermore, around 76.11 % of the Emiratis *H. pylori* positive participants were infected by the CagA positive strain. Further investigations might be needed in the future to confirm these results within a restricted Emiratis participants including children and adults. Among the African ethnicity, Ethiopians represented the highest prevalence to both *H. pylori* and CagA. These findings were previously reported by Khoder et al., in 2019 where Ethiopian babysitters also presented the highest *H. pylori* infection in a cross-sectional fecoprevalence study conducted on 350 participants.

In terms of ferritin and vitamin B12 deficiencies, our study found a significant association between *H. pylori* and ferritin deficiency only. However, the virulent oncoprotein CagA was found significantly correlated to both vitamin B12 and ferritin deficiencies. Several literature reviews have shown controversial studies related to *H. pylori*, ferritin and vitamin B12 deficiencies [[40], [41], [42],[44], [45], [46], [47], [48],[91], [92], [93], [94]] while few studies have explored the correlation between the oncoprotein CagA and ferritin, vitamin B12 deficiencies [43,95]. In the current study, the association between *H. pylori*, CagA, ferritin and vitamin B12 in the UAE population was investigated for the first time on a large scale. In UAE, studies assessing the prevalence of vitamin B12 deficiency are scarce. Only one recent study has revealed significant alterations of the vitamin B12 serum levels on a restricted population of one-year laparoscopic sleeve gastrectomy (LSG) patients. Vitamin B12 deficiency was assessed in 28 % of the patients compared to normal (5 %) (p < 0.001) [96]. Similarly, very few studies have assessed the ferritin deficiency in the UAE population. A study conducted in 2013 in UAE involving 394 participants revealed noteworthy findings. Among the women included in the study, it was observed that 16 % had iron deficiency anemia while 65 % of the women exhibited low ferritin values, with levels below 30 μg/L [97]. In our study, the prevalence of vitamin B12 and ferritin deficiencies was assessed to 35.4 % and 6.2 %, respectively. Even though this was not the main objective of our study, these findings were obtained for the first time and further studies need to be conducted to assess the deficiencies of those serum biomarkers into more oriented studies due to their implications in various health conditions.

Ferritin, a key protein involved in iron storage and homeostasis, has gained significant attention in clinical research due to its diverse implications in various health conditions. Alterations in ferritin levels have been associated with a wide range of conditions, including iron deficiency anemia, chronic inflammation, and certain chronic diseases [98,99]. Emerging evidences and several studies have suggested potential link between *H. pylori* and ferritin levels through multiple mechanisms. Firstly, *H. pylori*-associated chronic inflammation can lead to impaired iron absorption in the stomach, resulting in reduced iron availability for the synthesis of ferritin. Secondly, *H. pylori*-induced gastritis may disrupt the normal functioning of gastric parietal cells, which are involved in the release of gastric acid and intrinsic factor necessary for efficient iron absorption [46,100]. Consequently, impaired iron absorption can contribute to reduced ferritin levels [[101], [102], [103], [104]]. Furthermore, *H. pylori* infection has been associated with an increased risk of developing iron deficiency anemia, further emphasizing the potential association between *H. pylori,* and altered ferritin status [46]. However, it is important to note that the relationship between *H. pylori* and ferritin is complex and influenced by various factors such as individual host characteristics, coexisting conditions, and the specific *H. pylori* strain. It has been found that the presence of CagA positive *H. pylori* strains may influence ferritin levels in infected individuals. CagA has been shown to interact with cellular signaling pathways and disrupt normal cellular processes, including iron metabolism. Experimental studies have demonstrated that CagA can interfere with iron uptake and storage mechanisms in host cells, leading to alterations in ferritin levels [12]. Furthermore, CagA has been found to promote chronic inflammation and oxidative stress, both of which can affect iron homeostasis and contribute to changes in ferritin levels. In our study, statistically significant association has been found between *H. pylori*, CagA and ferritin deficiency. The specific mechanisms through which CagA influences ferritin regulation are still being investigated, but it is believed that CagA may play a role in the disruption of cellular iron metabolism. However, further research is needed to fully understand the complex interplay between *H. pylori*, CagA and ferritin and its implications in *H. pylori*-related diseases and iron-related disorders. Understanding the association between ferritin and *H. pylori* infection may contribute to improved management approaches for individuals affected by *H. pylori*-related gastrointestinal disorder.

On the other hand, *H. pylori* has been proposed to interfere with the absorption of vitamin B12, also known as cobalamin, which is an essential micronutrient with diverse roles in numerous physiological processes. Its clinical significance lies in its vital functions, including red blood cell production, nervous system maintenance, and DNA synthesis [105]. Deficiency of vitamin B12 can lead to various health complications, such as megaloblastic anemia, neuropathy, and cognitive impairment [105]. The malabsorption of the vitamin B12 induced by *H. pylori* seems to be related to an impaired production of the intrinsic factor [106] which is produced by gastric parietal cells to bind to vitamin B12, allowing its absorption in the terminal part of the small intestine [107]. *H. pylori-*associated gastritis can disrupt the normal functioning of these parietal cells, leading to a decreased intrinsic factor production. Consequently, individuals infected with *H. pylori* may experience impaired absorption of vitamin B12, resulting in lower levels of this essential vitamin. Therefore, it is crucial to consider the potential effects of *H. pylori* infection on vitamin B12 levels and to monitor individuals with *H. pylori*-related gastritis for vitamin B12 deficiency, ensuring accurate diagnosis and appropriate treatment. In our study, no statistical difference was observed between *H. pylori* and vitamin B12. However, a significant association was particularly found between the oncoprotein CagA and vitamin B12. Our findings are in agreement with several studies which have reported no correlation between *H. pylori* and vitamin B12 [42,108,109]. Rassol et al., reported no impact of *H. pylori* on the vitamin B12 level, folate and homocysteine levels [42]. Likewise, *H. pylori* infection was not recognized as a risk factor for low vitamin B12 levels in alcohol-dependent patients or in rural Mexican women [108,109]. However, it was reported by Abu Hilu et al., that *H. pylori* was negatively correlated to serum levels of vitamin B12 and may contribute to this deficiency [94]. Furthermore, there is a growing evidence suggesting a potential link between CagA protein of *H. pylori* and vitamin B12 levels [43,110]. Our results were aligned with similar study conducted on Turkish population where vitamin B12 deficiency was positively correlated with CagA positivity [43]. However, it's important to note that the link between CagA and Vitamin B12 levels is complex and influenced by other factors, such as the host's genetic predisposition and the presence of other coexisting conditions. Further research is needed to better understand the underlying mechanisms and clinical implications of the interaction between CagA and vitamin B12 in *H. pylori* infection.

The study offers a detailed overview of the prevalence of *H. pylori* and the oncoprotein CagA in the Sharjah Emirate, UAE. It takes into account the four major ethnicities, the multinational resident population, and various sociodemographic factors associated with the infection. Furthermore, the assessment of *H. pylori* and its virulent oncoprotein CagA and their association with ferritin and vitamin B12 deficiencies have been investigated for the first time. These data can strengthen national efforts aimed at preventing and eradicating *H. pylori*, ultimately reducing the complications and outcomes associated with the infection, such as GC. Understanding the link between *H. pylori* infection, CagA, and deficiencies in ferritin and vitamin B12 may enhance management strategies for individuals with *H. pylori*-related gastrointestinal disorders. Additional large-scale and multicenter epidemiological studies are being planned across the seven Emirates of the UAE to further this research.

## 5 Conclusions

Epidemiological studies conducted on *H. pylori* have underscored its pivotal role in evidence-based decision-making for the development and implementation of comprehensive management strategies. This is especially crucial in reducing the considerable burden of *H. pylori* infection and its associated adverse outcomes, notably GC. Effective eradication strategies demand current, location-specific data on *H. pylori* prevalence and its contributing factors. For the first time in the Sharjah Emirate, UAE, this study assessed the prevalence of *H. pylori*, the presence of its carcinogenic virulent factor CagA, and their associated risk factors including vitamin B12 and ferritin deficiencies among 1094 healthy asymptomatic adults. Strikingly, nearly half of the study participants tested positive for *H. pylori*, with over half of these cases involving the more virulent CagA positive strains. Notably, among Emirati participants, 76.11 % of those with *H. pylori* infection were CagA positive. Statistical analysis demonstrated a significant association between *H. pylori* infection and CagA status with gender, ethnicity, occupation, as well as deficiencies in ferritin and vitamin B12. These findings underscore the importance to prompt *H. pylori* detection and eradication, not only as a preventive measure against GC but also as an effective strategy to mitigate adverse effects on ferritin and vitamin B12 deficiencies, thereby enhancing overall health outcomes for individuals affected by *H. pylori* infection. The insights from this study will contribute to the establishment of accurate prevalence estimates of *H. pylori* and inform the development of efficient future national interventions and strategies.

## Funding

This research was funded by targeted research grant (1801110229) and competitive research grant (2201110266) at 10.13039/100016714University of Sharjah.

## Ethics declaration

The study received review and approval from the Research and Ethics Committee of the University of Sharjah under reference number REC-17-04-17-01. All participants provided informed consent to participate in the study.

## Data availability Statement

Data associated with the study has not been deposited into a publicly available repository. Data will be made available on request.

## CRediT authorship contribution statement

**Om Kolthoom M. Weisy:** Writing – review & editing, Investigation, Formal analysis. **Reena A. Kedia:** Methodology, Investigation, Formal analysis. **Ibrahim Mahmoud:** Writing – review & editing, Validation, Formal analysis. **Raed O. Abu Odeh:** Writing – review & editing, Resources. **Bashair M. Mussa:** Writing – review & editing, Resources, Methodology. **Salah Abusnana:** Writing – review & editing, Resources, Methodology. **Sameh S.M. Soliman:** Writing – review & editing, Methodology, Funding acquisition. **Jibran Sualeh Muhammad:** Writing – review & editing, Validation, Investigation. **Mohamad Hamad:** Writing – review & editing, Investigation. **Rose Ghemrawi:** Writing – review & editing, Resources, Methodology. **Ghalia Khoder:** Writing – review & editing, Writing – original draft, Resources, Project administration, Methodology, Funding acquisition, Formal analysis, Conceptualization.

## Declaration of competing interest

The authors declare that they have no known competing financial interests or personal relationships that could have appeared to influence the work reported in this paper.
